# Effect of conservation agriculture on soil fungal diversity in rice-wheat-greengram cropping system in eastern Indo-Gangetic plains of South Asia

**DOI:** 10.3389/fmicb.2024.1441837

**Published:** 2024-10-15

**Authors:** Rakesh Kumar, Jaipal Singh Choudhary, Sushanta Kumar Naik, Janki Sharan Mishra, Sushmita Banra, Shish Pal Poonia, Surajit Mondal, Anup Das, Karnena Koteswara Rao, Virender Kumar, Bhagwati Prasad Bhatt, Suresh Kumar Chaudhari, Ram Kanwar Malik, Andrew McDonald

**Affiliations:** ^1^ICAR Research Complex for Eastern Region, Patana, India; ^2^ICAR Research Complex for Eastern Region, Farming System Research Centre for Hill and Plateau Region, Ranchi, India; ^3^ICAR Directorate of Weed Research, Jabalpur, India; ^4^University Department of Zoology, Ranchi University, Morabadi, Ranchi, India; ^5^Cereal Systems Initiative for South Asia (CSISA)-CIMMYT, Patna, India; ^6^International Rice Research Institute, Los Banos, Philippines; ^7^Natural Resource Management Division, ICAR Krishi Anusandhan Bhawan-II, New Delhi, India; ^8^Soil and Crop Sciences Section, School of Integrative Plant Sciences, Cornell University, Ithaca, NY, United States

**Keywords:** soil fungal diversity, sustainable agriculture, soil parameters, Illumina MiSeq, zero-tillage

## Abstract

**Introduction:**

Conservation agriculture (CA) is emerging as an eco-friendly and sustainable approach to food production in South Asia. CA, characterized by reduced tillage, soil surface cover through retaining crop residue or raising cover crops, and crop diversification, enhances crop production and soil fertility. Fungal communities in the soil play a crucial role in nutrient recycling, crop growth, and agro-ecosystem stability, particularly in agricultural crop fields.

**Methods:**

This study investigates the impact of seven combinations of tillage and crop residue management practices of agricultural production systems, including various tillage and crop residue management practices, on soil fungal diversity. Using the Illumina MiSeq platform, fungal diversity associated with soil was analysed.

**Results and discussion:**

The results show that the partial CA-based (pCA) production systems had the highest number of unique operational taxonomic units (OTUs) (948 OTUs) while the conventional production system had the lowest number (665 OTUs). The major fungal phyla identified in the topsoil (0–15 cm) were Ascomycota, Basidiomycota, and Mortierellomycota, with their abundance varying across different tillage-*cum*-crop establishment (TCE) methods. Phylum Ascomycota was dominant in CA-based management treatments (94.9±0.62), followed by the partial CA (pCA)-based treatments (91.0 ± 0.37). Therefore, CA-based production systems play a crucial role in shaping soil fungal diversity, highlighting their significance for sustainable agricultural production.

## Introduction

Ensuring sustainable food production and meeting the growing demand for food underscores the essential role of soil health in agricultural success (Mondal et al., 2020). Soil adaptability is a critical component of sustainable agricultural production systems. However, plethora of soil tests and recommendations for best management practices can present challenges in the selection and interpretation, often failing to address specific needs. Soil parameters such as temperature, aeration, water content, and the interactions among the biotic and abiotic components play a crucial role (Kladivko, 2001). Specific soil management strategies within agroecosystems, such as reduced tillage, no-tillage, minimum tillage crop rotations, intercropping, and soil protection through mulching or retaining crop residue, can significantly enhance soil fertility and agricultural crop yields (Scopel et al., 2013). CA, which integrates these practices, is recognized as a cost-effective, sustainable, and environment-friendly alternative to the conventional production system (Hobbs et al., 2008). Implementing an effective soil conservation method, including maintaining the soil structure, minimizing the tillage, optimizing fertilizer use, conserving crop biomass, and diversifying crop yields, is crucial for sustainable agriculture (Mondal et al., 2021; Kumar et al., 2021).

The impact of climate change, increased tillage intensity, and greater agricultural demands will exacerbate soil degradation in the future (Mishra et al., 2022). Conventional agricultural practices such as excessive tillage, inadequate crop residue management, overreliance on chemical fertilizers, and insufficient crop rotations contribute significantly to soil degradation (Samal et al., 2017). Soil microbial populations are profoundly affected by alterations in these parameters, as the physico-chemical properties of the soil are modified during the conventional tillage processes, consequently impacting soil microbial diversity (Xin et al., 2018; Choudhary et al., 2020; Sun et al., 2018).

Microbes are integral components of soil biology, and the microbial communities in soil, particularly those in the rhizosphere, play a vital role in plant development. They enhance soil fertility, promote mineral solubility for better nutrient availability, and aid in plant adaptation to various biotic and abiotic factors (Khoshru et al., 2020; Mitra et al., 2021). The rhizosphere is inhabited by numerous microbial communities, both beneficial and detrimental to plant development (Panneerselvam et al., 2021; Mitra et al., 2022). Beneficial microorganisms enable plants to tolerate environmental changes, thereby increasing plant fitness in adverse conditions (Hassani et al., 2018; Lyu et al., 2021).

Fungi, a significant group of microorganisms found in the rhizosphere, play essential roles as biocontrol agents, biostimulants, and decomposers (Sagarika et al., 2022). After bacteria, fungi are the most abundant microorganisms on earth and in the soil (Purvis and Hector, 2000). Acquiring insight into the influence of various agricultural management strategies on fungal composition is crucial for enhancing soil resilience to environmental variations and promoting agricultural sustainability in specific geographic areas (Yazdani et al., 2009). Understanding the relationship between agriculture practices, types of fungi, populations of earthworms, and characteristics of soil offers helpful insight to improve agricultural management techniques to enhance food security and environmental sustainability in the Eastern Indo-Gangetic plains (Kumar et al., 2023).

Various global research projects have prioritized examining the impact of tillage, crop establishment, and crop residue on the taxonomic diversity of bacteria. However, they frequently neglected to investigate the structure and abundance of the soil fungal communities (Wang et al., 2017; Xia et al., 2019; Sun et al., 2018; Kumar et al., 2023). While previous research has primarily investigated the soil bacterial community structure in the context of conservation agricultural-based management practices, there has been a limited focus on fungal diversity and abundance within these practices. Therefore, exploring soil fungal communities is crucial for sustainability of agricultural production systems. Analysing the diversity of fungal communities in agricultural soil through culture-dependent methods is challenging. Hence, metagenomic approaches are necessary to understand the diversity and composition of fungi in agroecosystems. In this study, we investigated the important soil fungal communities present in the Eastern-Indo-Gangetic Plains (EIGP) and assessed the effects of different conservation agricultural-based management practices on the soil characteristics, earthworm abundance, and fungal community structure. We hypothesize that the implementation of the conservation agriculture-based production systems will alter the soil fungal diversity and abundance in rice-wheat-greengram cropping system in eastern Indo-Gangetic plains of India.

## Materials and methods

### Site description and experimental design

A long-term experiment was initiated in 2015 at the research farm of ICAR-Research Complex for Eastern Region, Patna, Bihar, India (25°24.912’ N and 85°03.536′ E). The experiments were conducted using a randomized block design, with three replications of seven treatments (T) or “scenarios” (Sc). The treatments included conventional or farmers’ practices and conservation agriculture based various tillage-*cum*-crop establishment (TCE) strategies for the rice-wheat-greengram cropping system. The farmers’ practices were characterized by the traditional/conventional tilling followed by puddling in rice, traditional/conventional tilling in wheat, and zero-tilling in mung bean. The detailed descriptions of each treatment and TCE method are provided in the Supplementary Table S1. Average annual weather details are presented in Supplementary Figure S1 for the experiment location.

### Soil sampling, soil parameters analysis, and earthworm count

Detailed methodologies for soil sampling and earthworm counting are described in Kumar et al. (2023). In brief, to create a typical composite sample, soils were taken from the top 0–15 cm of each plot at five randomly selected locations, and mixed. After seven years of experimentation, soil samples were collected under aseptic conditions after the wheat crop harvest in April 2022. A portion of composite sample was subjected to DNA extraction, while the remaining subsample underwent physico-chemical property analysis after being air-dried and sieved through a 2-mm screen. The pH and electrical conductivity (EC) were assessed for sample–water mixtures at a 1:2.5 ratio. Organic carbon (OC) content was measured using the dichromate oxidation method followed by titration with ferrous ammonium sulfate, as described by Walkley and Black (1934). The content of mineralisable nitrogen (N) was determined through the Kjeldahl method, with subsequent titration using diluted sulfuric acid, based on the protocol by Subbiah and Asija (1956). Available phosphorus (P) was measured using the NaHCO3-ascorbic acid method, according to Watanabe and Olsen (1965), and available potassium (K) was determined with the ammonium acetate method using a flame photometer, as outlined by Hanway and Heidel (1952). Available micronutrient content was determined using the DTPA (Diethylenetriaminepentaacetic acid) extraction method as described by Lindsay and Norvell (1978). Earthworm populations were counted in the early morning hours using the standard methodology outlined by Fonte et al. (2009).

### Genomic DNA extraction and amplicon sequencing

Genomic DNA was extracted from processed soil samples using the Alexgen Soil DNA Extraction Kit following the manufacturer’s protocol. Ultrapure water (without DNA from samples) was used to exclude the possibility of false-positive PCR results as a negative control. The quality of the extracted DNA was evaluated using 0.8% gel electrophoresis, while the DNA concentration was quantified using a Qubit 4.0 fluorometer (Supplementary Table S2). The internal transcribed spacer (ITS) region of the fungal communities was amplified using the primer pair ITS3-F: 5′ TCGTCGGCAGCGTC AGATGTGTATAAGAGACAGGCATCGATGAAGAACGCAGC 3′ and ITS4-R: 5′ GTCTCGTGGGCTCGGAGATGTGTATAA GAGACAGTCCTCCGCTTATTGATATGC 3′ (White et al., 1990). The PCR protocol included an initial denaturation phase at 95°C for 10 min, followed by 30 cycles of denaturation at 95°C for 60 s, annealing at 56°C for 60 s, and extension at 72°C for 90 s. A final extension phase was conducted at a temperature of 72°C for 10 min. The PCR products were purified using Ampure XP beads (Agencourt Bioscience, Beverly, MA, United States) and quantified using a qPCR quantification kit for the Illumina sequencing platform. Dual index adapters were attached using the Nextera indices kit (Illumina, San Diego, CA, United States). Amplicons were assessed for integrity using an Agilent tape station and quantified using a Qubit Fluorometer 4.0 with the Qubit 1xdsDNA HS assay kit (Invitrogen technologies). Paired-end sequencing was performed using 2 × 300 bp on the Illumina Miseq platform (Illumina, San Diego, CA, United States), with three replicates per treatment and a total of 21 samples sequenced.

### Microbiome data analyses

Raw data sequences underwent quality control, including removal of low-quality bases and adapters, using TrimGalore V0.4.0 (Krueger et al., 2021). Forward and reverse sequences were merged into single sequences using PEAR v. 0.9.6 (Zhang et al., 2014). Processed and merged reads were then analyzed using the Quantitative Insights Into Microbial Ecology 2 (QIIME 2) pipeline v. 2020.8 (Bolyen et al., 2019). Sequence reads were quality filtered and denoised to obtain amplicon sequence variants (ASVs), with UNITE ITS reference (99% clustered ASVs from UNITE v. 8.0) used as a positive filter to characterize sequences as ITS (UNITE Community, 2019). Taxonomic classification of each ASV against ITS was performed using the feature-classifier-classify-sklearn, a pre-fitted sklearn-based taxonomy classifier.

### Soil quality index

The Soil Quality Index (SQI) was established by the application of non-linear techniques (Bastida et al., 2006). The minimum dataset (MDS) was established using principal component analysis (PCA), a multivariate statistical method, by utilizing parameters that showed significant differences (Andrews et al., 2002). The system attributes were deemed to be most accurately represented by principal components (PCs) with eigenvalues ≥1 and higher factor loadings. Still, in this investigation, the eigenvalues of the first three principal components (PCs) exceeded 1, thus accounting for only 86% of the total variance. To account for over 90% of the variance, we selected PCs with eigenvalues greater than 0.9, which accounted for over 5% of the total variance in the calculation of the soil quality index. We selected highly weighted factors from each PC that had eigenvalues larger than 0.40 or absolute values within 10% of the highest factor loading. When multiple factors were retained from a single PC, we conducted a multivariate correlation analysis. Only factors with correlation coefficients less than 0.60 were included in the MDS. We utilised non-linear methods to designate scores to various parameters after selecting all the MDS soil quality indicators, as illustrated by the subsequent equation (Bastida et al., 2006). For all MDS indicators, we implemented a “more is better” approach, except for ECe, which was subjected to a “less is better” function.


(1) $$S=\frac{a}{\left(1+{\left(\frac{x}{x_{0}}\right)}^{b}\right)}$$

The maximum value (which in our case is 1) that the function can attain is denoted by a in this equation. $x_{0}$ is the mean of all observations for that parameter across each treatment, x represents the value of an individual observation for the corresponding parameter, and b represents the slope value (−2.5) of the equation (Bastida et al., 2006). The sum of the weighted scores of the MDS parameters for each observation was used to calculate the SQI using Equation 2.


(2) $$SQI=\overset{n}{\underset{i=1}{\sum }}W_{i}S_{i}$$

Where ‘W’ represents the weightage of the MDS variable obtained from PCA, and ‘S’ represents the score of that specific variable derived from Equation 1.

### Statistical analyses

Downstream analysis was conducted using the online MicrobiomeAnalyst web platform^1^ and R tools 4.0.4. Data were filtered based on a minimum count of four per library before statistical analysis. The Bray–Curtis dissimilarity index was employed to calculate the Alpha diversity indices (Ace, Chao1, Shannon, and Simpson) (Dhariwal et al., 2017). Box plots for alpha diversity indices were generated using the vegan package in the R environment. The VennDiagram package in R was used to visualize the relationship between unique and common operational taxonomic units (OTUs) among different treatments (Chen and Boutros, 2011). The Bray–Curtis dissimilarity index and principal coordinate analysis (PCoA) were employed to analyze fungal beta diversity among treatments. Analysis of variance (ANOVA) was performed to assess the impact of different treatments on fungal population diversity. Different fungal diversity indices were categorized using the Kruskal-Wallis H test in SPSS (version 22.0). Pearson’s correlation test and principal component analysis (PCA) were used to assess the impact of soil characteristics on fungal diversity, conducted using Microsoft Excel (XLStat 7.5, Addinsoft).

## Results

### Composition and diversity of fungal communities in different tillage-*cum*-crop establishment (TCE) methods

**Figure 1:**
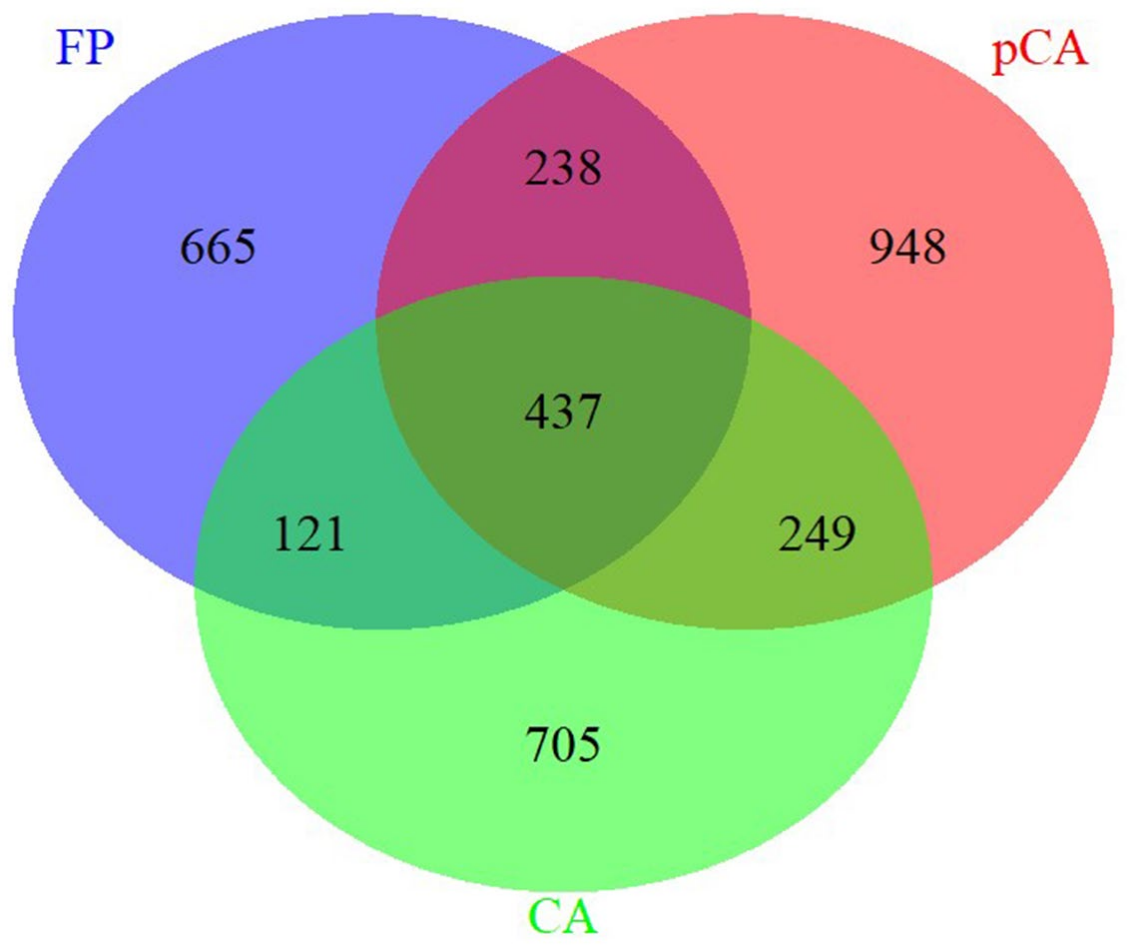
Unique and shared operational taxonomic units (OUTs) illustrated by Venn diagram across various conservation agriculture-based production systems.

The ITS region amplicon was sequenced on the Illumina MiSeq platform for seven different treatments, each consisting of three replicates. The rarefaction curve analysis indicated that all samples reached saturation, indicating suitability for further analyses. At a similarity threshold of 97%, a total of 437 operational taxonomic units (OTUs) were shared between conservation and farmer-based agricultural management practices (Figure 1). The identified fungi were classified into four major phyla: Ascomycota, Basidiomycota, Mortierellomycota, Mucoromycota, and one unidentified phylum of fungi.

**Figure 2:**
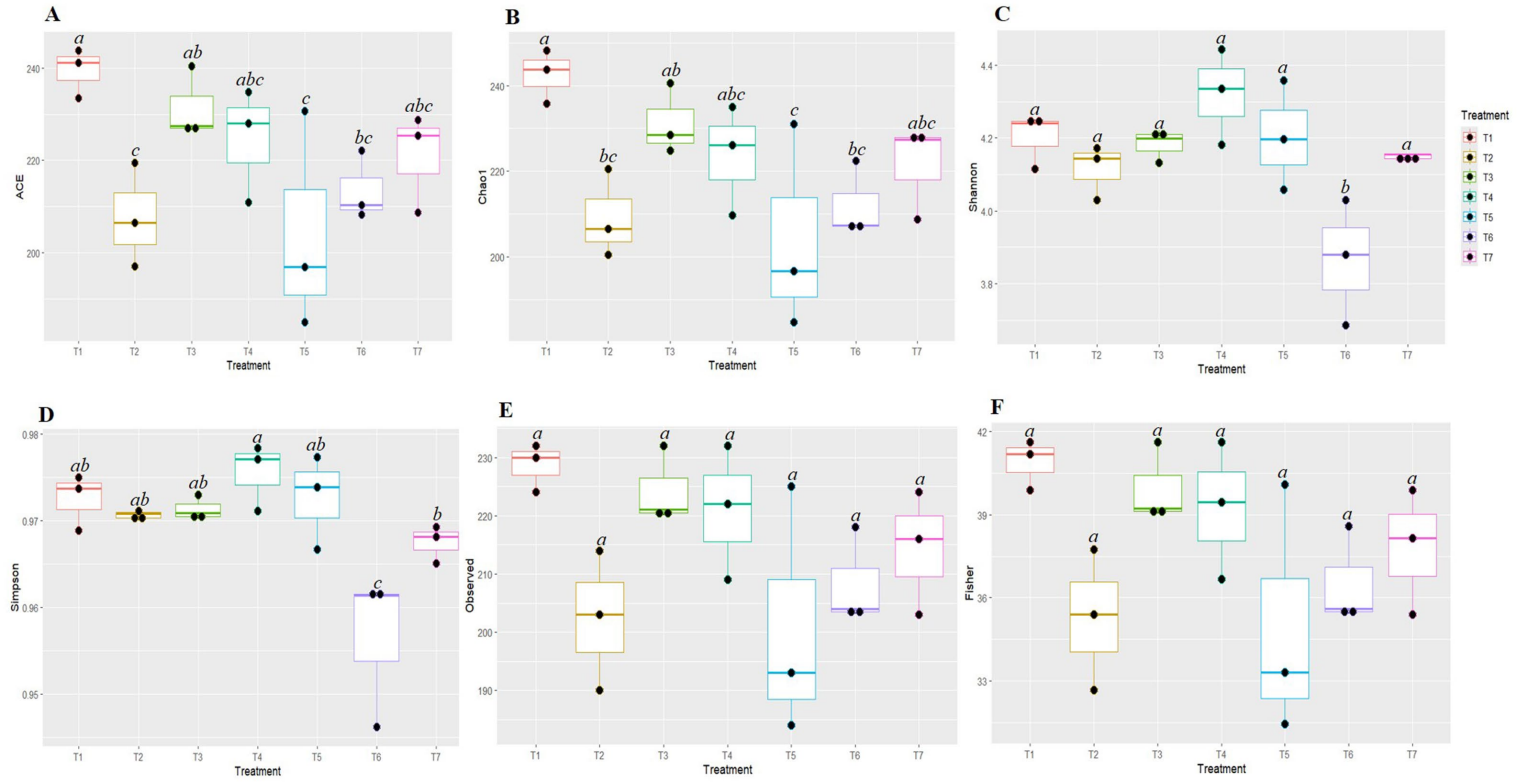
Box plots represent different diversity indices (Ace **(A)**, Chao1 **(B)**, Shannon **(C)**, Simpson **(D)**, Observed **(E)** and Fisher **(F)**) among conservation agriculture-based production systems. Alphabets above box indicate significant differences among agricultural practices, (*p* < 0.05). Colors denote the different treatments on experiment.

The Kruskal-Wallis H test was employed to evaluate the significance of differences in the alpha diversity of fungal communities among the various tillage-cum-crop establishment (TCE) methods (Figure 2). The species richness indices Ace and Chao1 were significantly highest in FP (T1) (239.427 and 242.637, respectively) and lowest in pCA (T5) (204.175 and 204.133, respectively). The diversity indices based on Shannon and Simpson were highest in T4 (4.32 and 0.976) and the lowest in T6 (3.865 and 0.956), respectively. No significant differences were observed in the observed indices and Fisher diversity indices among different production scenarios. Therefore, the highest species richness was observed in T1, while the highest fungal diversity was observed under T4.

**Figure 3:**
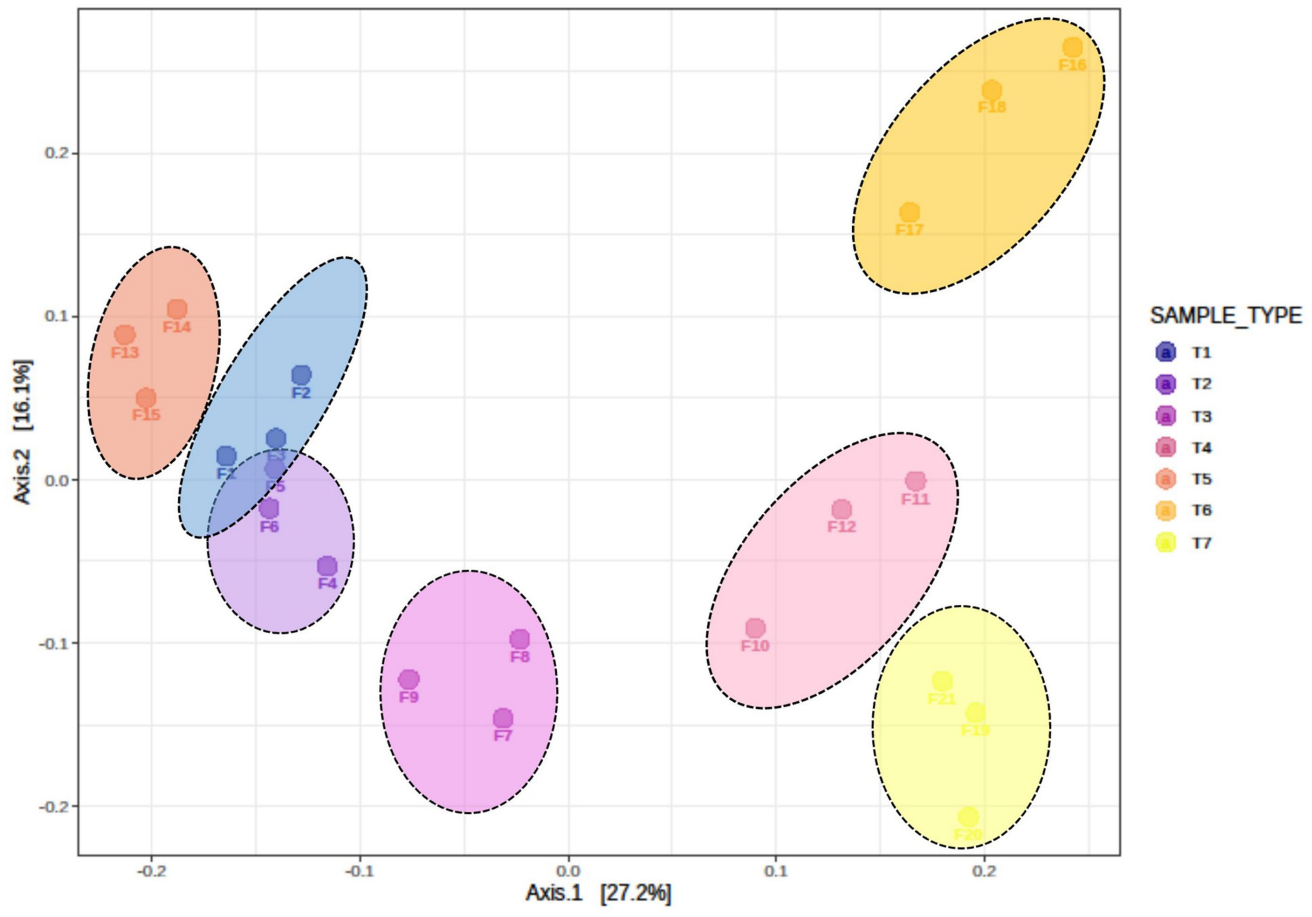
Beta diversity, as illustrated by principal coordinate analysis (PCoA) based on the Bray–Curtis dissimilarity index, indicates significant differences in fungus composition among different treatments of conservation agriculture-based production systems (ANOSIM R = 0.70909, *p* < 0.001). T1-First treatment, T2-Second treatment, T3-Third treatment, T4-Fourth treatment, T5-Fifth treatment, T6-Sixth treatment, T7-Seventh treatment.

Beta diversity among the different treatments was assessed using Bray–Curtis dissimilarity index and visualized through principal coordinate analysis (PCoA) (Figure 3). The analysis revealed significant differences in fungal communities among different scenarios (*R* = 0.70909, *p* < 0.001). Beta diversity results indicated that the fungal communities of T1 and T2 (farmers’ practices) clustered and overlapped, indicating shared the fungal communities. In contrast, fungal communities of T3, T4, T5, T6, and T7 clustered separately and were distinct from each other. These results confirm that different scenarios harbour significantly different fungal communities.

At the phylum level, the most abundant fungal phyla observed were Ascomycota, Basidiomycota, Mortierellomycota, Mucoromycota, and an unidentified phylum group (Supplementary Figure S2). Among these, Ascomycota was the most abundant fungal phylum across all the scenarios. Specifically, the phylum Ascomycota was highest in T7 (94.88%) and the lowest in T5 (91.00%). In contrast, Basidiomycota showed the highest abundance in T5 (3.38%) and the lowest in T7 (0.68%). Both Mucoromycota and the unidentified fungi phylum were less abundant across all scenarios.

**Figure 4:**
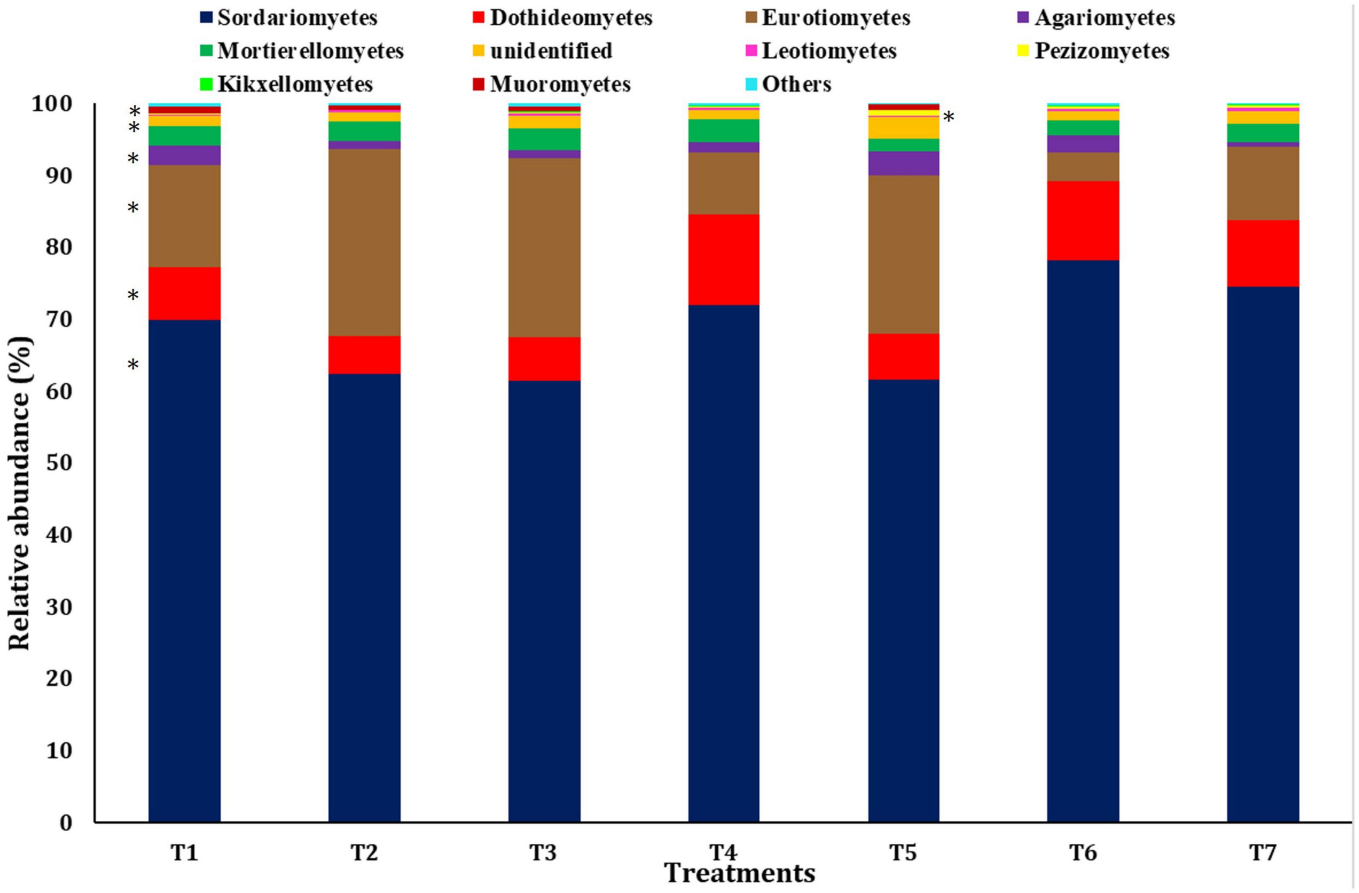
Relative abundance of dominant fungus at the class level among different treatments of conservation agriculture-based production system. The significant difference is indicated by ‘*’.

A bar diagram was employed to explain the significant differences in the relative abundance at the class level (Figure 4). The most abundant class observed was Sordariomycetes of the phylum Ascomycota across all scenarios, followed by Euriomycetes and Dothideomycetes, both belonging to the phylum Ascomycota. Sordariomycetes were the most abundant in T6 (78.23 ± 1.39%), followed by T7 (74.55 ± 1.71%). The abundance of Euriomycetes was highest in scenarios employing farmer practices and pCA-based production (25.96 ± 2.21% and 24.85 ± 2.87% in T2 and T3, respectively), and the lowest abundance was observed in CA-based treatments. Conversely, Dothideomycetes showed the highest abundance in CA-based production scenarios (12.70 ± 1.70% in T4) and the lowest in scenarios employing farmer practices (5.25 ± 0.24% in T2). No significant differences were observed in the abundance of classes Mortierellomycetes, Leotiomycetes, Kickxellomycetes, and other grouped classes.

**Figure 5:**
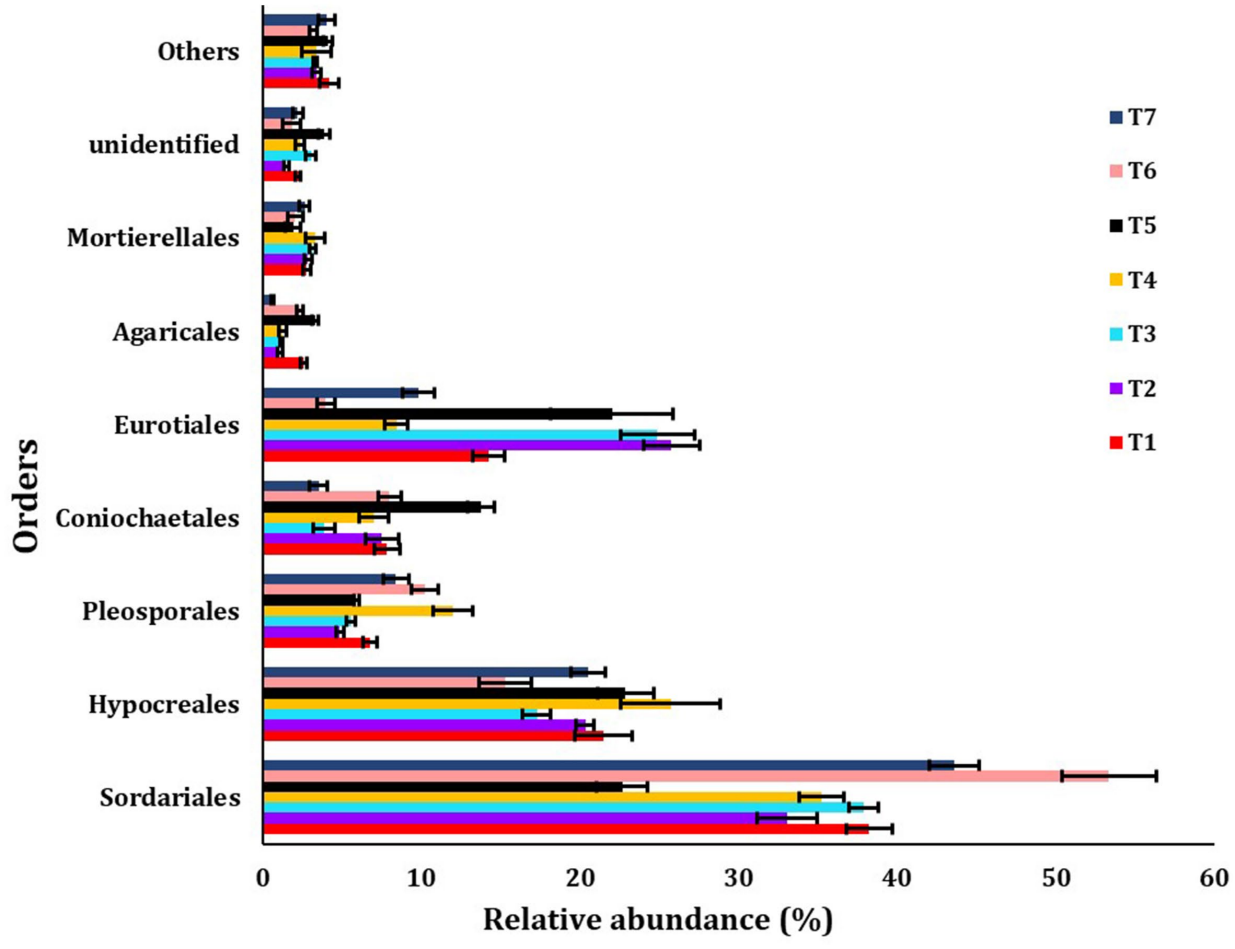
The relative abundance of dominanting order among different treatments of conservation agriculture-based production systems. The standard errors of the mean are represented by whiskers.

The major orders identified were Sordariales, Hypocreales, Pleosporales, Coniochaetales, Eurotiales, Agaricales, Mortierellales, unidentified, and others (Figure 5). The most abundant fungus order observed was Sordariales across all scenarios, with the highest abundance found in T6 (53.33%) and the lowest in T5 (22.61%).

**Figure 6:**
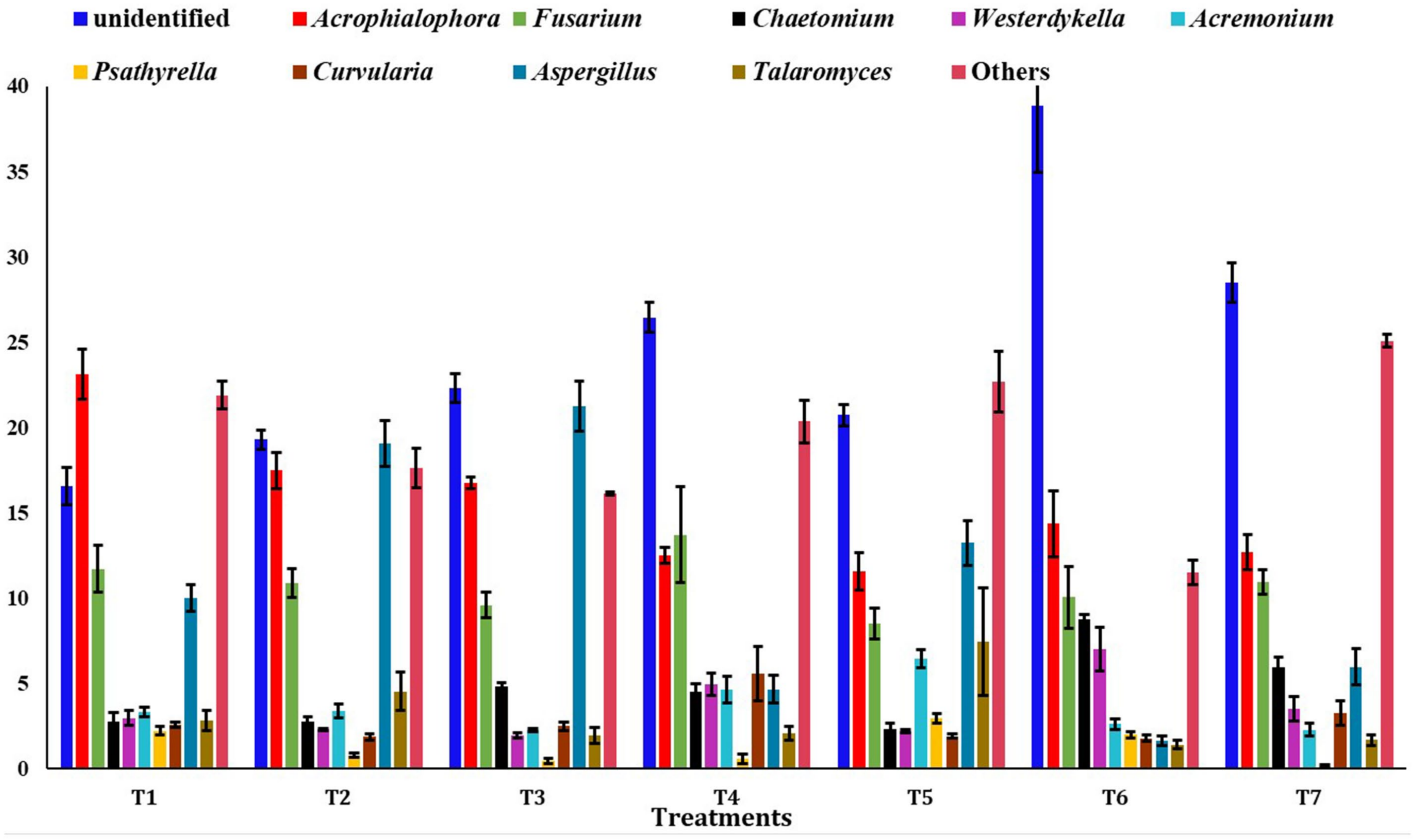
Relative abundance of dominating genus among different treatments of conservation agriculture-based production system. The standard errors of mean (SEm) are represented by whiskers.

At the genus level, the unidentified fungi were highly abundant across all scenarios (T2: 19.30%, T3: 22.31%, T4: 26.45%, T5: 20.72%, T6: 38.85%, and T7: 28.49%), except in T1 (16.5%) (Figure 6). In T1, the most abundant genus was *Acrophialophora* (23.14%). Other major fungal genera identified included *Fusarium*, *Aspergillus*, *Chaetomium*, *Westerdykella*, *Acremonium*, *Psathyrella*, *Curvularia*, and *Talaromyces.*

### Soil quality parameters and earthworm activity and its impact on fungal communities

A comparative assessment of soil quality parameters across various management scenarios indicated that agricultural practices have a significant impact on soil properties (Table 1). While differences in soil pH were not significant among the scenarios, the conservation agriculture (CA)-based production scenarios (T4 and T7) exhibited significantly higher electrical conductivity (EC) compared to other partial CA-based management practices and traditional/conventional farmer practices. The highest soil organic carbon (SOC) content (9.65 g kg^−1^) was observed in the CA-based production scenario (T4), outperforming others. Available nitrogen (N) content was significantly greater in the CA-based management scenarios (T4 and T7) compared to traditional/conventional practices. Both CA and partial CA scenarios had significantly more available potassium (K) than traditional practices, with the highest available K content (182.2 kg ha^−1^) recorded in CA-based production scenario (T7). Micronutrients such as manganese (Mn), zinc (Zn), and copper (Cu) were higher in CA scenario (T7), with contents of 38.6, 10.9, and 9.9 mg kg^−1^, respectively than others.

**Table 1:** Soil chemical properties and earthworm count in different scenarios of agricultural management.

Treatments	pH	EC (dS m^−1^)	SOC (g kg^−1^)	N (kg ha^−1^)	P (kg ha^−1^)	K (kg ha^−1^)	Fe (mg kg^−1^)	Mn (mg kg^−1^)	Zn (mg kg^−1^)	Cu (mg kg^−1^)	Earthworm counts (no. m^−3^)
T1	7.34^a^	0.12^b^	8.62^c^	212.9^ab^	23.5^c^	128.5^d^	155.6^a^	31.1^bc^	10.40^a^	7.10^c^	343^e^
T2	7.42^a^	0.10^b^	8.81^bc^	204.3^b^	27.2^ab^	129.8^d^	142.1^a^	29.3^c^	8.27^c^	6.82^c^	625^d^
T3	7.24^a^	0.13^b^	9.45^ab^	227.4^ab^	26.0^bc^	144.1^cd^	156.1^a^	38.3^a^	9.40^abc^	8.03^bc^	766^c^
T4	7.34^a^	0.11^b^	8.90^bc^	237.1^ab^	28.0^ab^	169.4^ab^	156.4^a^	31.0^bc^	9.80^abc^	9.63^a^	1024^b^
T5	7.49^a^	0.25^a^	9.65^a^	243.8^a^	29.1^ab^	156.4^bc^	147.1^a^	35.2^ab^	9.93^ab^	9.20^ab^	1130^a^
T6	7.13^a^	0.15^b^	9.05^abc^	236.5^ab^	28.7^ab^	161.9^abc^	157.7^a^	36.5^a^	8.53^bc^	9.21^ab^	802^c^
T7	7.24^a^	0.30^a^	9.36^ab^	247.4^a^	29.7^a^	182.2^a^	155.6^a^	38.7^a^	10.87^a^	9.90^a^	660^d^

Means value followed by same superscript letters within each coloumn are not statistically different (Duncan’s multiple range test; *p* ≤ 0.05).

**Figure 7:**
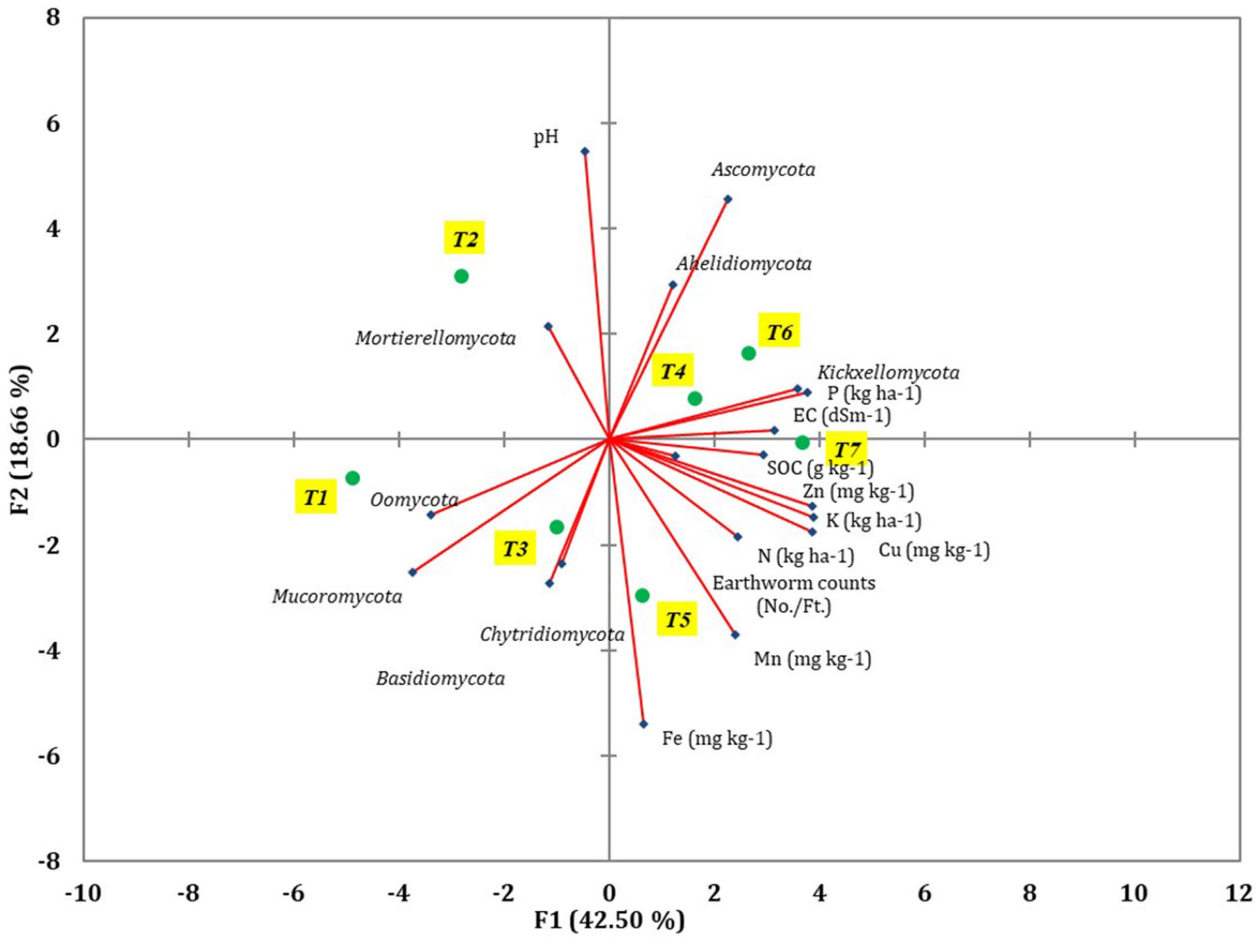
The different soil parameters, major fungal phyla, and earthworm count were revealed using PCA coordinates of different treatments of conservation agriculture-based production systems.

The diversity of fungal community was influenced by the soil characteristics and earthworm populations, as revealed by principal component analysis (PCA) (Figure 7). Various soil characteristics, earthworm populations, and major fungal phyla were used to construct the PCA to assess the most important factors impacting fungal communities (Supplementary Table S3). Out of the total variance, 86.14% was accounted for by four principal components with eigenvalues greater than 0.9. PC1 and PC2 contributed 42.5 and 18.66% to the total variance, respectively, while PC3 and PC4 contributed 14.87 and 10.11%, respectively. In PC1, EC, SOC, N, P, K, Cu, Mucoromycota, Oomycota, and Kickxellomycota were highly loaded. Among the 21 variables analyzed, pH, N, P, K, Fe, Cu, Ascomycota, and Mortierellomycota were influenced by tillage-cum-crop establishment (TCE).

**Figure 8:**
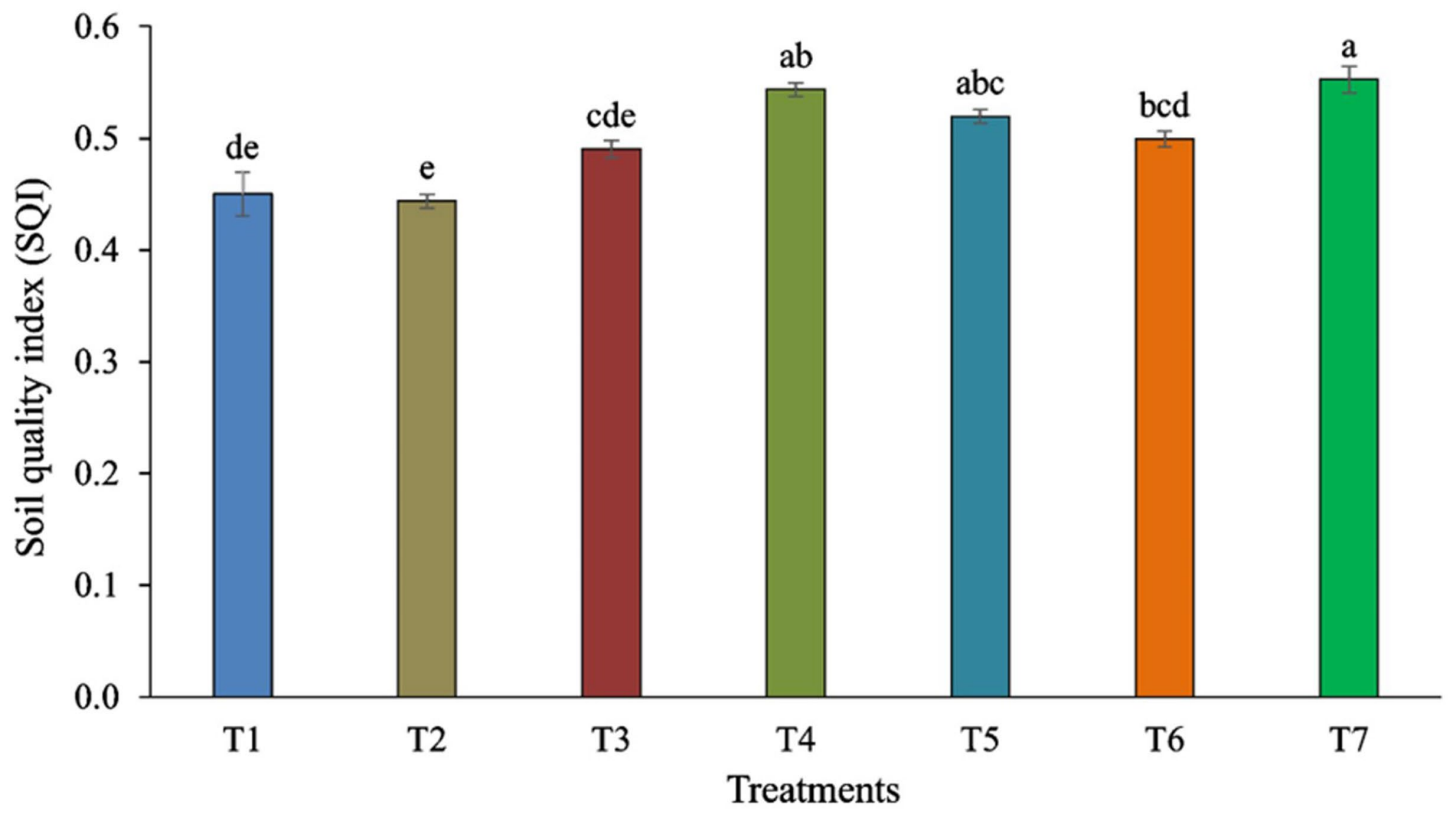
Soil Quality Index (SQI) is influenced by different tillage and crop establishment scenarios. Standard errors of the mean are represented by vertical bars. Different small letters indicate significant differences at *p* ≤ 0.05.

Pearson’s test was conducted to investigate the correlation between earthworm count, soil characteristics, and major fungal phyla to minimize redundancy in the PCA. pH showed a positive correlation with the phylum Aphelidiomycota (*r* = 0.63) (Table 2). EC exhibited a negative correlation with Mucoromycota (*r* = −0.62). No significant correlation was observed between Fe, Mn, Zn, and different fungal phyla. Soil organic carbon (SOC) was negatively correlated with the phylum Oomycota (*r* = −0.73). N was negatively correlated with the phyla Mucoromycota (*r* = −0.72) and Oomycota (*r* = −0.62), while it was positively correlated with the phylum Kickxellomycota (*r* = 0.77). P was negatively associated with the phylum Mucoromycota (*r* = −0.88) but positively associated with Kickxellomycota (*r* = 0.80). The K showed a negative correlation with Mucoromycota (*r* = −0.79) and Oomycota (*r* = −0.65), while it was positively correlated with Kickxellomycota (*r* = 0.79). The Cu exhibited a negative correlation with Mucoromycota (*r* = −0.79) and Oomycota (*r* = −0.64), but it was positively correlated with Kickxellomycota (*r* = 0.85). The Soil Quality Index (SQI) varied significantly across different treatments. The highest SQI value of 0.55 was observed in T7, which was significantly higher than in T1, T2, T3, and T6. However, it was similar to the SQI values of T4 and T5 (Figure 8).

**Table 2:** Inter-relationship between different soil parameters, earthworm count, and major fungus phyla in different treatments of conservation agriculture-based production system.

Fungus phyla	pH	EC (dS m^−1^)	SOC (g kg^−1^)	N (kg ha^−1^)	P (kg ha^−1^)	K (kg ha^−1^)	Fe (mg kg^−1^)	Mn (mg kg^−1^)	Zn (mg kg^−1^)	Cu (mg kg^−1^)
Earthworm counts (No./Ft.)	−0.38	−0.02	0.28	0.54	**0.63**	0.57	0.27	0.26	−0.41	**0.65**
Ascomycota	0.45	0.49	0.36	0.28	0.55	0.41	−0.46	0.01	0.26	0.31
Basidiomycota	−0.09	−0.21	−0.18	−0.04	−0.24	−0.27	0.17	−0.09	−0.19	−0.11
Mortierellomycota	0.21	−0.32	−0.26	−0.37	−0.41	−0.20	−0.02	−0.31	0.21	−0.30
Mucoromycota	−0.24	**−0.62**	−0.50	**−0.72**	**−0.88**	**−0.79**	0.16	−0.18	−0.23	**−0.79**
Oomycota	−0.05	−0.45	**−0.73**	**−0.62**	−0.77	**−0.65**	0.11	−0.44	0.00	**−0.64**
Kickxellomycota	0.08	0.38	0.43	**0.77**	**0.80**	**0.79**	0.08	0.16	0.09	**0.85**
Chytridiomycota	−0.14	−0.31	0.36	−0.10	−0.37	−0.32	0.19	0.34	−0.20	−0.23
Ahelidiomycota	**0.64**	0.20	0.24	0.23	0.39	0.05	−0.52	−0.28	−0.12	0.20

Bold values represent a significant relationship at *p* < 0.05.

## Discussion

In this study, we investigated that the changes in fungal diversity structure following the implementation of long-term tillage-cum-establishment methods in Eastern Indo-Gangetic Plains (EIGP) alluvial soils within a sub-tropical humid environment in the eastern region. Although extensive research exists on bacterial diversity in soil habitats, there is limited knowledge on fungal communities in agricultural soil (Anderson et al., 2003; Buée et al., 2009; Curlevski et al., 2010; Fierer et al., 2007; Lynch and Thorn, 2006; Stromberger, 2005; Kumar et al., 2023). Agricultural management practices, SOC, and enzyme activity alter microbial diversity in the soil (Govaerts et al., 2007). Comparatively high fungal diversity was observed under conservation agricultural (CA)-based management practices, possibly due to the continuous presence of crop residues and reduced soil-surface disturbance (Choudhary et al., 2018). Zero tillage with residue cover may support fungal growth and activity by facilitating the development and maintenance of large hyphal networks. Previous reports suggest that zero-tillage practices lead to changes in the fungal communities, resembling those of the natural ecosystems, and significantly contribute to decomposition and nutrient cycling (Bailey et al., 2002).

Our study found that predominant phylum was Ascomycota, followed by Basidiomycota in all scenarios, consistent with previous studies (Miura et al., 2015; Choudhary et al., 2018). The relatively high nitrogen concentrations in the soils likely contributed to the abundance of Ascomycota. Previous studies have reported that Ascomycota can utilize resources in adverse environmental conditions by tolerating stressful situations such as inadequate nutrient availability (Chen et al., 2017). Basidiomycota plays a crucial role in decomposing plant debris in soil, particularly in litter rich in lignin, which was relatively higher in partial CA treatments with retained and incorporated crop residues in our study (Liwanag et al., 2014).

The predominant classes were Sordariomycetes, Dothideomycetes, and Eurotiomycetes, all belonging to the phylum Ascomycota and predominant in conservation agricultural practices. This pattern aligns with the previous studies (Wang et al., 2017; Choudhary et al., 2018). Sordariomycetes produce cellulolytic enzymes and are typically found in soils rich in crop residues, as observed in our study (Phosri et al., 2012). The class Dothideomycetes of the phylum Ascomycota significantly differed among scenarios and was more abundant in CA-based management practices. Dothideomycetes play an important role in the breakdown of complex polysaccharides like cellulose in dead or partially digested plant tissues (Hyde et al., 2013).

Our study found that the predominant order was Sordariales, followed by Hypocreales, a result consistent with previous research conducted in residue-rich and less disturbed soils (Klaubauf et al., 2010; Choudhary et al., 2018). Fungi belonging to this order are a source of secondary metabolites.

The identified predominant genera were *Acrophialophora*, *Fusarium*, *Acremonium*, and *Aspergillus*. Fungal communities are essential for soil fertility as they affect nutrient absorption efficiency and crop susceptibility to pathogens in paddy fields (Giraldo et al., 2019). *Fusarium*, for instance, is known to cause mycetoma, leading to osteolytic lesions, soft tissue swelling, and abnormalities (Tomimori-Yamashita et al., 2002). Fungi affect ecosystem functions, such as plant health, and are thus an important part of soil biodiversity (Fisher et al., 2012; Duniere et al., 2017; Wang et al., 2017). The Sordariales and Hypocreales orders were more dominant in full CA-based management scenarios (T6 and T4 scenarios), possibly due to differences in available carbon sources and nitrogen availability in those CA-based scenarios (Miura et al., 2015).

Our studies revealed that different soil components such as pH, SOC, N, P, K, and Cu significantly influence the diversity of fungal communities. Changes in the physio-chemical qualities of the soil may be linked to long-term shifts in the structure of the fungal community. According to the ecological guild analysis, Cu interacted negatively with the pathotroph communities, whereas Ca interacted positively with symbiotroph, endophyte, and saprotroph groups (Panneerselvam et al., 2023). Soil fungal diversity was affected by various soil components. The proper functioning and quality of the soil ecosystem depend on the diversity of soil microbes (Garbeva et al., 2004; Liu et al., 2015; Ding et al., 2017). The most influential soil parameters for maintaining better soil quality of CA-based production system were DTPA-extractable Cu, soil pH, Mortierellomycota, DTPA-extractable-Zn. The higher SQI in scVII was attributed to better soil chemical and biological properties confined to higher DTPA-extractable Cu, DTPA-extractable-Zn, Mortierellomycota, and optimum pH. Persistent alterations in composition of fungal communities may be associated with changes in the physical and chemical properties of the soil. Hgher concentration of SOC in the soil results in an elevation in CO_2_ levels in the *soil*.

## Conclusion

Fungal diversity was significantly influenced by long-term tillage and crop establishment (TCE) methods, where partial or full CA-based management practices showed high fungal diversity. Soil fungal communities significantly varied in different TCE at the phylum, class, and order levels. Major phyla reported in the top soil (0–15 cm) were Ascomycota, Basidiomycota, and Mortierellomycta, but their abundance varied in different TCE management methods, with soil dominated by the phylum Ascomycota. Our study showed that soil organic carbon, chemical properties, and earthworm populations were significantly influenced by the TCE, ultimately affecting fungal diversity. The results of this study could be used as the basis for further research into environmentally friendly farming practices. Finally, it is important to connect the taxonomic and functional analyses of soil microbes to learn more about the impact of farming on soil characteristics and the ecological functions that microbes perform.

## Data Availability

The datasets presented in this study can be found in online repositories. The names of the repository/repositories and accession number(s) can be found at: https://www.ncbi.nlm.nih.gov/bioproject/PRJNA1101727.

## Footnotes

^1^ https://www.microbiomeanalyst.ca

## References

[ref1] Anderson I. C. Campbell C. D. Prosser J. I. (2003). Potential bias of fungal 18S rDNA and internal transcribed spacer polymerase chain reaction primers for estimating fungal biodiversity in soil. Environ. Microbiol. 5, 36–47. doi: 10.1046/j.1462-2920.2003.00383.x, PMID: 12542711

[ref2] Andrews S. S. Karlen D. L. Mitchell J. P. (2002). A comparison of soil quality indexing methods for vegetable production systems in northern California. Agric. Ecosyst. Environ. 90, 25–45. doi: 10.1016/S0167-8809(01)00174-8

[ref3] Bailey V. L. Smith J. L. Bolton H. (2002). Fungal-to-bacterial ratios in soils investigated for enhanced C sequestration. Soil Biol. Biochem. 34, 997–1007. doi: 10.1016/S0038-0717(02)00033-0

[ref4] Bastida F. Moreno J. L. Hernández T. García C. (2006). Microbiological degradation index of soils in a semiarid climate. Soil Biol. Biochem. 38, 3463–3473. doi: 10.1016/j.soilbio.2006.06.001

[ref5] Bolyen E. Rideout J. R. Dillon M. R. Bokulich N. A. Abnet C. C. Al-Ghalith G. A.. (2019). Reproducible, interactive, scalable and extensible microbiome data science using QIIME 2. Nat. Biotechnol. 37, 852–857. doi: 10.1038/s41587-019-0209-9, PMID: 31341288 PMC7015180

[ref6] Buée M. Reich M. Murat C. Morin E. Nilsson R. H. Uroz S.. (2009). 454 pyrosequencing analyses of forest soils reveal an unexpectedly high fungal diversity. New Phytol. 184, 449–456. doi: 10.1111/j.1469-8137.2009.03003.x 19703112

[ref7] Chen H. Boutros P. C. (2011). VennDiagram: a package for the generation of highly-customizable Venn and Euler diagrams in R. BMC Bioinformatics 12:35. doi: 10.1186/1471-2105-12-35, PMID: 21269502 PMC3041657

[ref8] Chen Y. L. Xu T. L. Veresoglou S. D. Hu H. W. Hao Z. P. Hu Y. J.. (2017). Plant diversity represents the prevalent determinant of soil fungal community structure across temperate grasslands in northern China. Soil Biol. Biochem. 110, 12–21. doi: 10.1016/j.soilbio.2017.02.015

[ref9] Choudhary M. Jat H. S. Datta A. Sharma P. C. Rajashekar B. Jat M. L. (2020). Topsoil bacterial community changes and nutrient dynamics under cereal based climate-smart Agri-food systems. Front. Microbiol. 11:542545. doi: 10.3389/fmicb.2020.01812 PMC7399647 32849419

[ref10] Choudhary M. Jat H. S. Datta A. Yadav A. K. Sapkota T. B. Mondal S.. (2018). Sustainable intensification influences soil quality, biota, and productivity in cereal-based agroecosystems. Appl. Soil Ecol. 126, 189–198. doi: 10.1016/j.apsoil.2018.02.027

[ref11] Curlevski N. J. A. Xu Z. H. Anderson I. C. Cairney J. W. (2010). Diversity of soil and rhizosphere fungi under *Araucaria bidwillii* (bunya pine) at an Australian tropical montane rainforest site. Fungal Divers. 40, 12–22. doi: 10.1007/s13225-009-0001-0

[ref12] Dhariwal A. Chong J. Habib S. King I. L. Agellon L. B. Xia J. (2017). MicrobiomeAnalyst: a web-based tool for comprehensive statistical, visual and meta-analysis of microbiome data. Nucleic Acids Res. 45, W180–W188. doi: 10.1093/nar/gkx295 28449106 PMC5570177

[ref13] Ding J. Jiang X. Guan D. Zhao B. Ma M. Zhou B.. (2017). Influence of inorganic fertilizer and organic manure application on fungal communities in a long-term field experiment of Chinese Mollisols. Appl. Soil Ecol. 111, 114–122. doi: 10.1016/j.apsoil.2016.12.003

[ref14] Duniere L. Xu S. Long J. Elekwachi C. Wang Y. Turkington K.. (2017). Bacterial and fungal core microbiomes associated with small grain silages during ensiling and aerobic spoilage. BMC Microbiol. 17, 1–16. doi: 10.1186/s12866-017-0947-0 28253864 PMC5335695

[ref16] Fierer N. Breitbart M. Nulton J. Salamon P. Lozupone C. Jones R.. (2007). Metagenomic and small-subunit rRNA analyses reveal the genetic diversity of bacteria, archaea, fungi, and viruses in soil. Appl. Environ. Microbiol. 73, 7059–7066. doi: 10.1128/AEM.00358-07, PMID: 17827313 PMC2074941

[ref17] Fisher M. C. Henk D. A. Briggs C. J. Brownstein J. S. Madoff L. C. McCraw S. L.. (2012). Emerging fungal threats to animal, plant and ecosystem health. Nature 484, 186–194. doi: 10.1038/nature10947, PMID: 22498624 PMC3821985

[ref18] Fonte S. J. Winsome T. Six J. (2009). Earthworm populations in relation to soil organic matter dynamics and management in California tomato cropping systems. Appl. Soil Ecol. 41, 206–214. doi: 10.1016/j.apsoil.2008.10.010

[ref19] Garbeva P. Van Veen J. A. Van Elsas J. D. (2004). Microbial diversity in soil: selection of microbial populations by plant and soil type and implications for disease suppressiveness. Annu. Rev. Phytopathol. 42, 243–270. doi: 10.1146/annurev.phyto.42.012604.135455 15283667

[ref20] Giraldo A. Hernandez-Restrepo M. Crous P. W. (2019). New plectosphaerellaceous species from Dutch garden soil. Mycol. Prog. 18, 1135–1154. doi: 10.1007/s11557-019-01511-4

[ref21] Govaerts B. Mezzalama M. Unno Y. Sayre K. D. Luna-Guido M. Vanherck K.. (2007). Influence of tillage, residue management, and crop rotation on soil microbial biomass and catabolic diversity. Appl. Soil Ecol. 37, 18–30. doi: 10.1016/j.apsoil.2007.03.006

[ref22] Hanway J. J. Heidel H. (1952). Soil analysis methods as used in Iowa state college, soil testing laboratory. Iowa Agric. 54, 1–31.

[ref23] Hassani M. A. Durán P. Hacquard S. (2018). Microbial interactions within the plant holobiont. Microbiome 6, 1–17. doi: 10.1186/s40168-018-0445-0 29587885 PMC5870681

[ref24] Hobbs P. R. Sayre K. Gupta R. (2008). The role of conservation agriculture in sustainable agriculture. Philos. Trans. R. Soc. B Biol. Sci. 363, 543–555. doi: 10.1098/rstb.2007.2169, PMID: 17720669 PMC2610169

[ref25] Hyde K. D. Jones E. G. Liu J. K. Ariyawansa H. Boehm E. Boonmee S.. (2013). Families of Dothideomycetes. Fungal Divers. 63, 1–313. doi: 10.1007/s13225-013-0263-4

[ref26] Khoshru B. Mitra D. Khoshmanzar E. Myo E. M. Uniyal N. Mahakur B.. (2020). Current scenario and future prospects of plant growth-promoting rhizobacteria: an economic valuable resource for the agriculture revival under stressful conditions. J. Plant Nutr. 43, 3062–3092. doi: 10.1080/01904167.2020.1799004

[ref27] Kladivko E. J. (2001). Tillage systems and soil ecology. Soil Tillage Res. 61, 61–76. doi: 10.1016/S0167-1987(01)00179-9

[ref28] Klaubauf S. Inselsbacher E. Zechmeister-Boltenstern S. Wanek W. Gottsberger R. Strauss J.. (2010). Molecular diversity of fungal communities in agricultural soils from Lower Austria. Fungal Divers. 44, 65–75. doi: 10.1007/s13225-010-0053-1 23794962 PMC3688302

[ref29] Krueger F. James F. Ewels P. Afyounian E. Schuster-Boeckler B. (2021). FelixKrueger/TrimGalore: v0.6.7: Zendo. doi: 10.5281/zenodo.7598955

[ref30] Kumar R. Choudhary J. S. Naik S. K. Mondal S. Mishra J. S. Poonia S. P.. (2023). Influence of conservation agriculture-based production systems on bacterial diversity and soil quality in rice-wheat-greengram cropping system in eastern indo-Gangetic Plains of India. Front. Microbiol. 14:1181317. doi: 10.3389/fmicb.2023.1181317, PMID: 37485518 PMC10356824

[ref31] Kumar R. Mishra J. S. Mondal S. Meena R. S. Sundaram P. K. Bhatt B. P.. (2021). Designing an ecofriendly and carbon-cum-energy efficient production system for the diverse agroecosystem of South Asia. Energy 214:118860. doi: 10.1016/j.energy.2020.118860

[ref32] Lindsay W. L. Norvell W. (1978). Development of a DTPA soil test for zinc, iron, manganese, and copper. Soil Sci. Soc. Am. J. 42, 421–428. doi: 10.2136/sssaj1978.03615995004200030009x

[ref33] Liu J. Sui Y. Yu Z. Shi Y. Chu H. Jin J.. (2015). Soil carbon content drives the biogeographical distribution of fungal communities in the black soil zone of Northeast China. Soil Biol. Biochem. 83, 29–39. doi: 10.1016/j.soilbio.2015.01.009

[ref34] Liwanag A. de Vries R. P. Benoit I. Unlocking the lignin degrading potential of ascomycete fungi. (2014). Available at: https://www.aspergillus.org.uk/content/unlocking-lignin-degrading-potential-ascomycete-fungi. (Accessed April 05, 2022).

[ref35] Lynch M. D. Thorn R. G. (2006). Diversity of basidiomycetes in Michigan agricultural soils. Appl. Environ. Microbiol. 72, 7050–7056. doi: 10.1128/AEM.00826-06 16950900 PMC1636180

[ref36] Lyu D. Zajonc J. Pagé A. Tanney C. A. Shah A. Monjezi N.. (2021). Plant holobiont theory: the phytomicrobiome plays a central role in evolution and success. Microorganisms 9:675. doi: 10.3390/microorganisms9040675, PMID: 33805166 PMC8064057

[ref37] Mishra J. S. Kumar R. Mondal S. Poonia S. P. Rao K. K. Dubey R.. (2022). Tillage and crop establishment effects on weeds and productivity of a rice-wheat-mungbean rotation. Field Crop Res. 284:108577. doi: 10.1016/j.fcr.2022.108577, PMID: 35924187 PMC9214547

[ref38] Mitra D. Djebaili R. Pellegrini M. Mahakur B. Sarker A. Chaudhary P.. (2021). Arbuscular mycorrhizal symbiosis: plant growth improvement and induction of resistance under stressful conditions. J. Plant Nutr. 44, 1993–2028. doi: 10.1080/01904167.2021.1881552

[ref39] Mitra D. Mondal R. Khoshru B. Senapati A. Radha T. K. Mahakur B.. (2022). Actinobacteria-enhanced plant growth, nutrient acquisition, and crop protection: advances in soil, plant, and microbial multifactorial interactions. Pedosphere 32, 149–170. doi: 10.1016/S1002-0160(21)60042-5

[ref40] Miura T. Niswati A. Swibawa I. G. Haryani S. Gunito H. Shimano S.. (2015). Diversity of fungi on decomposing leaf litter in a sugarcane plantation and their response to tillage practice and bagasse mulching: implications for management effects on litter decomposition. Microb. Ecol. 70, 646–658. doi: 10.1007/s00248-015-0620-9 25933637

[ref41] Mondal S. Mishra J. S. Poonia S. P. Kumar R. Dubey R. Kumar S.. (2021). Can yield, soil C and aggregation be improved under long-term conservation agriculture in the eastern indo-Gangetic plain of India? Eur. J. Soil Sci. 72, 1742–1761. doi: 10.1111/ejss.13092 34413692 PMC8359171

[ref42] Mondal S. Poonia S. P. Mishra J. S. Bhatt B. P. Rao K. K. Saurabh K.. (2020). Short-term (5 years) impact of conservation agriculture on soil physical properties and organic carbon in a rice-wheat rotation in indo-Gangetic plains of Bihar. Eur. J. Soil Sci. 71, 1076–1089. doi: 10.1111/ejss.12879

[ref43] Panneerselvam P. Senapati A. Chidambaranathan P. Prabhukarthikeyan S. R. Mitra D. Govindharaj G. P. P.. (2023). Long-term impact of pulses crop rotation on soil fungal diversity in aerobic and wetland rice cultivation. Fungal Biol. 127, 1053–1066. doi: 10.1016/j.funbio.2023.04.005, PMID: 37344007

[ref44] Panneerselvam P. Senapati A. Sharma L. Nayak A. K. Kumar A. Kumar U.. (2021). Understanding rice growth-promoting potential of *Enterobacter* spp. isolated from long-term organic farming soil in India through a supervised learning approach. Curr. Res. Microb. Sci. 2:100035. doi: 10.1016/j.crmicr.2021.100035, PMID: 34841326 PMC8610300

[ref45] Phosri C. Polme S. Taylor A. F. Koljalg U. Suwannasai N. Tedersoo L. (2012). Diversity and community composition of ectomycorrhizal fungi in a dry deciduous dipterocarp forest in Thailand. Biodivers. Conserv. 21, 2287–2298. doi: 10.1007/s10531-012-0250-1

[ref46] Purvis A. Hector A. (2000). Getting the measure of biodiversity. Nature 405, 212–219. doi: 10.1038/35012221 10821281

[ref47] Sagarika M. S. Parameswaran C. Senapati A. Barala J. Mitra D. Prabhukarthikeyan S. R.. (2022). Lytic polysaccharide monooxygenases (LPMOs) producing microbes: a novel approach for rapid recycling of agricultural wastes. Sci. Total Environ. 806:150451. doi: 10.1016/j.scitotenv.2021.150451, PMID: 34607097

[ref48] Samal S. K. Rao K. K. Poonia S. P. Kumar R. Mishra J. S. Prakash V.. (2017). Evaluation of long-term conservation agriculture and crop intensification in rice-wheat rotation of indo-Gangetic Plains of South Asia: carbon dynamics and productivity. Eur. J. Agron. 90, 198–208. doi: 10.1016/j.eja.2017.08.006, PMID: 29056851 PMC5637878

[ref49] Scopel E. Triomphe B. Affholder F. Da Silva F. A. M. Corbeels M. Xavier J.. (2013). Conservation agriculture cropping systems in temperate and tropical conditions, performances and impacts. A review. Agron. Sustain. Dev. 33, 113–130. doi: 10.1007/s13593-012-0106-9

[ref50] Stromberger M. E. (2005). “Fungal communities of agroecosystems” in The fungal community: Its organization and role in the ecosystem. eds. Dighton J. White J. F. Oudemans P.. 3rd ed (Boca Raton: CRC Press), 813–832.

[ref51] Subbiah B. V. Asija G. L. (1956). A rapid procedure for the estimation of available nitrogen in soils. Curr. Sci. 25, 259–260.

[ref52] Sun R. Li W. Dong W. Tian Y. Hu C. Liu B. (2018). Tillage changes vertical distribution of soil bacterial and fungal communities. Front. Microbiol. 9:347825. doi: 10.3389/fmicb.2018.02456, PMID: 29686662 PMC5900040

[ref53] Tomimori-Yamashita J. Ogawa M. M. Hirata S. H. Fischman O. Michalany N. S. Yamashita H. K.. (2002). Mycetoma caused by fusarium solani with osteolytic lesions on the hand: case report. Mycopathologia 153, 11–14. doi: 10.1023/A:1015294117574 11913759

[ref54] UNITE Community (2019). UNITE QIIME release for Fungi: UNITE Community. doi: 10.15156/BIO/786334

[ref55] Walkley A. Black I. A. (1934). An examination of the Degtjareff method for determining organic carbon in soils: effect of variations in digestion conditions and of inorganic soil constituents. Soil Sci. 63, 251–264. doi: 10.1097/00010694-194704000-00001

[ref56] Wang Y. Li C. Tu C. Hoyt G. D. DeForest J. L. Hu S. (2017). Long-term no-tillage and organic input management enhanced the diversity and stability of soil microbial community. Sci. Total Environ. 609, 341–347. doi: 10.1016/j.scitotenv.2017.07.053 28753509

[ref57] Watanabe F. S. Olsen S. R. (1965). Test of ascorbic acid method for determining phosphorus in water and sodium bicarbonate extracts of soil. Soil Sci. Soc. Am. Proc. 29, 677–678. doi: 10.2136/sssaj1965.03615995002900060025x

[ref58] White T. J. Bruns T. Lee S. Taylor J. (1990). Amplification and direct sequencing of fungal ribosomal RNA genes for phylogenetics, Innis M. A. Gelfand D. H. Sninsky J. J. White T. J.. (Eds.), PCR Protocols, Academic Press, Inc., San Diego, CA, pp. 315–322.

[ref9001] Xia X. Zhang P. He L. Gao X. Li W. Zhou Y.. (2019). Effects of tillage managements and maize straw returning on soil microbiome using 16S rDNA sequencing. J. Integr. Plant Biol. 61, 765–777. doi: 10.1111/jipb.12802 30912294

[ref59] Xin X. L. Yang W. L. Zhu Q. G. Zhang X. F. Zhu A. N. Zhang J. B. (2018). Abundance and depth stratification of soil arthropods as influenced by tillage regimes in a sandy loam soil. Soil Use Manag. 34, 286–296. doi: 10.1111/sum.12412

[ref60] Yazdani M. Bahmanyar M. A. Pirdashti H. Esmaili M. A. (2009). Effect of phosphate solubilization microorganisms (PSM) and plant growth promoting rhizobacteria (PGPR) on yield and yield components of corn (*Zea mays* L.). World Acad. Sci. Eng. Technol. 49:90e92. doi: 10.5281/zenodo.1080014

[ref61] Zhang J. Kobert K. Flouri T. Stamatakis A. (2014). PEAR: a fast and accurate Illumina paired-end reAd mergeR. Bioinformatics 30, 614–620. doi: 10.1093/bioinformatics/btt593 24142950 PMC3933873

