# Supplementary material for: Effect of conservation agriculture on soil fungal diversity in rice-wheat-greengram cropping system in eastern Indo-Gangetic plains of South Asia
Source: Front Microbiol. 2024 Oct 15;15:1441837. doi: 10.3389/fmicb.2024.1441837 (PMC11523130; doi:10.3389/fmicb.2024.1441837)
Supplement: Supplementary file 1 [file Data_Sheet_1.docx]

**Supplementary Table 1.** Tillage-cum-crop-establishments (TCE) drivers for agricultural change in different scenarios.

| Treatment notations | Tillage | | | Crop establishment | | | Residue management | | |
| --- | --- | --- | --- | --- | --- | --- | --- | --- | --- |
|  | Rice | Wheat | Mung bean | Rice | Wheat | Mung bean | Rice | Wheat | Mung bean |
| T1: RPTR-BCW-ZTM (FP) | Cultivator: 2 passes (dry tillage: DT)  Rotavator: 1 pass (wet tillage: WT) | Cultivator: 2 passes  Rotavator: 1 pass | Zero till | 25-days old seedlings, manually transplanted with random geometry | Broadcasting | Drill seeding with Happy Seeder | ~30% incorporated in soil | ~30% retained on soil surface | 100% incorporated |
| T2: LPTR-CTW-ZTM (FP) |  |  |  | 25-days old seedlings, manually transplanted in lines at 25 x 15 cm apart. | Drill seeding with Happy Seeder |  |  |  |  |
| T3: CTMTR-ZTW-ZTM (pCA) |  | Zero-till |  | 18-days old seedlings, machine transplanting at 23 x 14 cm apart. |  |  | ~30% retained on soil surface |  |  |
| T4: ZTMTR-ZTW-ZTM (CA) | Zero-till (flooding before transplanting) |  |  | 18-days old seedlings, machine transplanting at 23 x 14 cm apart. |  |  |  |  | 100% removed |
| T5: SRI-SWI-ZTM (pCA) | Cultivator: 2 passes (DT)  Rotavator: 1 pass (WT) | Cultivator: 2 passes  Rotavator: 1 pass |  | 12-days old seedlings, manual transplanting at 25 x 25 cm apart. | Manual seeding |  | ~30% incorporated in soil |  | 100% incorporated |
| T6: CTDSR-ZTW-ZTM (pCA) | Cultivator: 2 passes  Rotavator: 1 pass | Zero-till |  | Drill seeding at 22.5 cm row spacing | Drill seeding with Happy Seeder |  | ~30% retained on soil surface |  |  |
| T7: ZTDSR-ZTW-ZTM (CA) | Zero-till |  |  | Drill seeding at 22.5 cm row spacing |  |  |  |  | 100% retained on the soil surface |

**Supplementary Table 2. Quantification and quality check (QC) of library using Qubit Fluorometer**

| Treatments/sample ids | Concentration (ng/μl) | Volume (μl) | Yield (μg) | Remarks |
| --- | --- | --- | --- | --- |
| Sc1/F1 | 38.6 | 50 | 1.93 | QC pass |
| Sc1/F2 | 36.4 | 50 | 1.82 | QC pass |
| Sc1/F3 | 40.2 | 50 | 2.01 | QC pass |
| Sc2/F4 | 49.2 | 50 | 2.46 | QC pass |
| Sc2/F5 | 26.81 | 50 | 1.34 | QC pass |
| Sc2/F6 | 36.01 | 50 | 1.80 | QC pass |
| Sc3/F7 | 30.8 | 50 | 1.54 | QC pass |
| Sc3/F8 | 20.2 | 50 | 1.01 | QC pass |
| Sc3/F9 | 48.0 | 50 | 2.4 | QC pass |
| Sc4/F10 | 47.4 | 50 | 2.37 | QC pass |
| Sc4/F11 | 21.4 | 50 | 1.07 | QC pass |
| Sc4/F12 | 32.6 | 50 | 1.63 | QC pass |
| Sc5/F13 | 50.8 | 50 | 2.54 | QC pass |
| Sc5/F14 | 53.0 | 50 | 2.65 | QC pass |
| Sc5/F15 | 55.4 | 50 | 2.77 | QC pass |
| Sc6/F16 | 61.6 | 50 | 3.08 | QC pass |
| Sc6/F17 | 32.4 | 50 | 1.62 | QC pass |
| Sc6/F18 | 41.6 | 50 | 2.08 | QC pass |
| Sc7/F19 | 39.4 | 50 | 1.97 | QC pass |
| Sc7/F20 | 44.0 | 50 | 2.20 | QC pass |
| Sc7/F21 | 56.0 | 50 | 2.8 | QC pass |

**Supplementary Table 3.** Based on different soil parameters, fungal phyla and earthworm count, principle component, and factor loading were calculated.

| **Principle components** | **PC1** | **PC2** | **PC3** | **PC4** |
| --- | --- | --- | --- | --- |
| Eigenvalue | 8.0745 | 3.5447 | 2.8266 | 1.9216 |
| Variability (%) | 42.4974 | 18.6563 | 14.8770 | 10.1136 |
| Cumulative % | 42.4974 | 61.1537 | 76.0307 | 86.1443 |
| Factor loading | | | | |
| pH | -0.1127 | **0.8668** | -0.1487 | 0.1638 |
| EC (dSm^-1^) | **0.7549** | 0.0283 | 0.1758 | 0.5065 |
| SOC (g kg^-1^) | **0.7069** | -0.0482 | 0.1005 | -0.0868 |
| N (kg ha^-1^) | **0.9302** | -0.2806 | -0.0130 | 0.1632 |
| P (kg ha^-1^) | **0.9081** | 0.1423 | -0.2060 | -0.1207 |
| K (kg ha^-1^) | **0.9259** | -0.1999 | 0.1081 | 0.0771 |
| Fe (mg kg^-1^) | 0.1571 | **-0.8562** | 0.2440 | 0.1044 |
| Mn (mg kg^-1^) | 0.5768 | -0.5905 | 0.2823 | 0.0168 |
| Zn (mg kg^-1^) | 0.3021 | -0.0515 | 0.5091 | **0.7244** |
| Cu (mg kg^-1^) | **0.9361** | -0.2352 | -0.0343 | 0.0779 |
| Earthworm counts (No./Ft.^2^) | 0.5855 | -0.2953 | -0.4370 | -0.5660 |
| Ascomycota | 0.5404 | **0.7237** | 0.4110 | -0.0452 |
| Basidiomycota | -0.2729 | -0.4342 | **-0.7792** | 0.2854 |
| Mortierellomycota | -0.2763 | 0.3420 | **0.7794** | -0.2510 |
| Mucoromycota | **-0.8966** | -0.4019 | 0.0824 | -0.0017 |
| Oomycota | **-0.8116** | -0.2284 | -0.1921 | 0.4778 |
| Kickxellomycota | **0.8597** | 0.1542 | -0.2575 | -0.1754 |
| Chytridiomycota | -0.2161 | -0.3764 | 0.2084 | -0.5325 |
| Ahelidiomycota | 0.2931 | 0.4646 | **-0.7627** | 0.1789 |


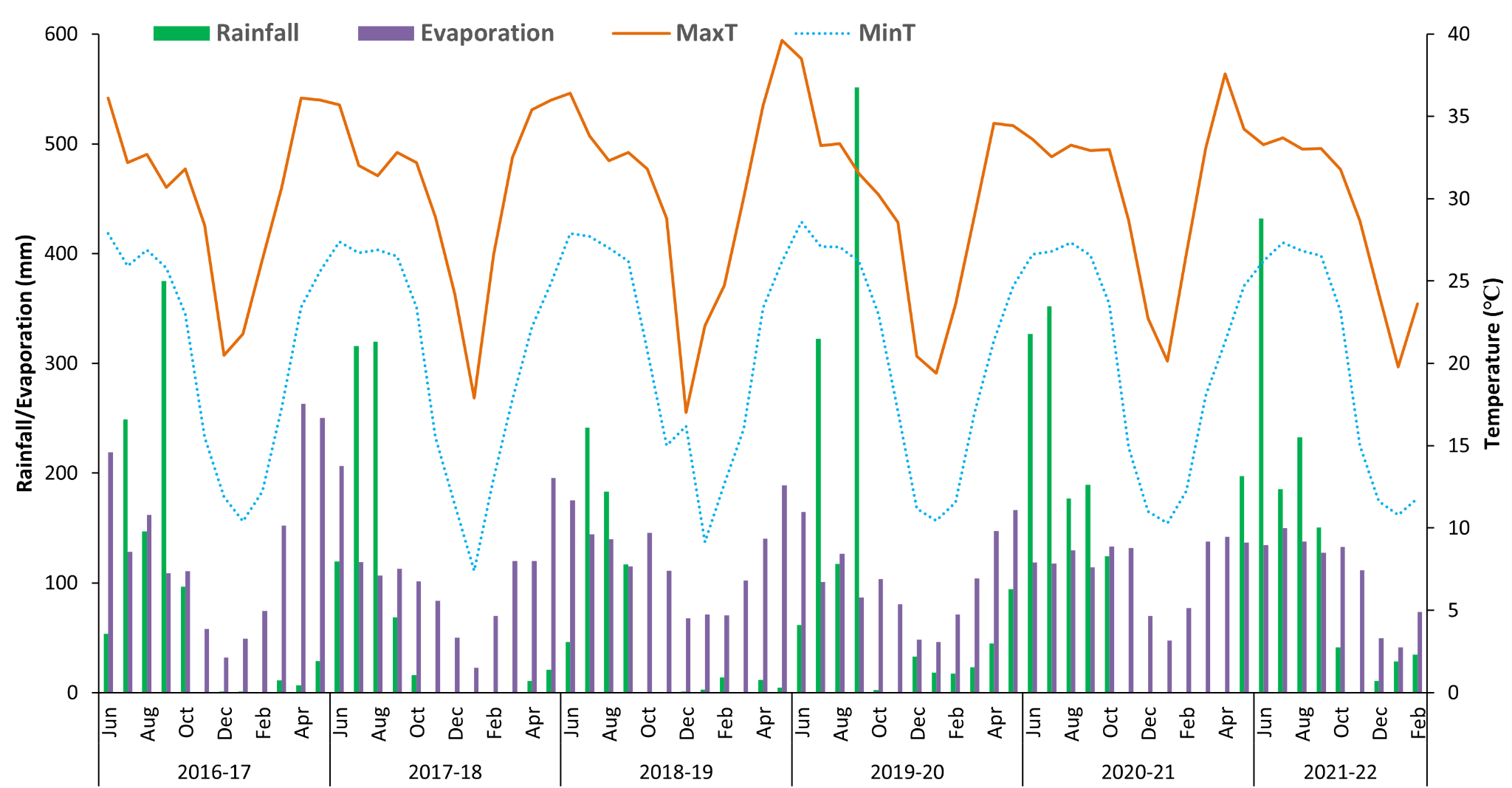


**Supplementary Figure 1.** Different weather parameters during experimentation at the experimental sites (2016-2022). [Max T and Min T: average maximum and minimum temperature (°C)]


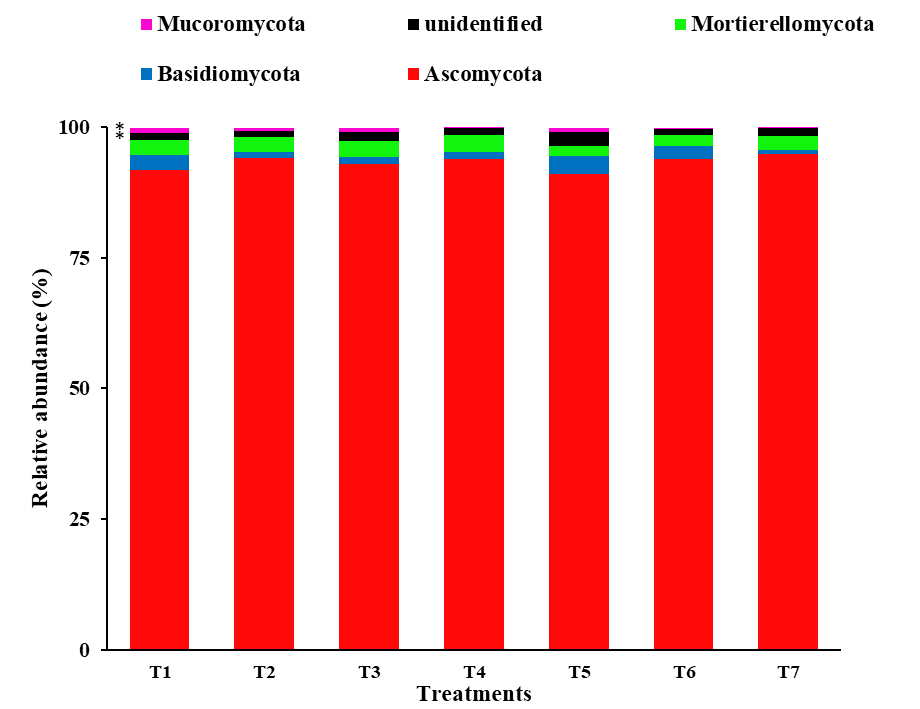


**Supplementary Figure 2.** Relative abundance of dominant fungus at phylum level between different treatments of conservation agriculture-based production system. The significant difference indicated by asterisk ‘*’ mark.
